# Endovascular treatment of cerebral venous sinus thrombosis in a thrombocytopenic patient with systemic lupus erythematosus and antiphospholipid syndrome: A CARE-compliant case report

**DOI:** 10.1097/MD.0000000000044961

**Published:** 2025-10-03

**Authors:** Yu Chen, Yen-Jun Lai, Ting-Hui Chang, Chien-Sheng Wu

**Affiliations:** a Division of Allergy, Immunology, and Rheumatology, Far Eastern Memorial Hospital, New Taipei City, Taiwan, ROC; b Department of Medical Imaging, Far Eastern Memorial Hospital, New Taipei City, Taiwan, ROC; c School of Medicine, Fu Jen Catholic University, New Taipei City, Taiwan, ROC.

**Keywords:** antiphospholipid syndrome, cerebral venous sinus thrombosis, endovascular intervention, magnetic resonance imaging, thrombocytopenia

## Abstract

**Rationale:**

The co-occurrence of extensive cerebral venous sinus thrombosis (CVST) and severe thrombocytopenia is rare even in patients with triple-positive antiphospholipid syndrome (APS) and systemic lupus erythematosus. This case illustrates the complexity of managing a thrombocytopenic patient with CVST and endovascular thrombectomy as a rescue therapy.

**Patient concerns:**

The patient was a 33-year-old female with newly diagnosed systemic lupus erythematosus and a 2-day history of fever, headache, and dizziness. During admission, right periorbital ecchymosis and thrombocytopenia (14,000/μL) were observed, and the patient was diagnosed with triple-positive APS. The patient complained of new-onset blurred vision, impaired right lateral gaze. Suspecting increased intracranial pressure, brain magnetic resonance imaging (MRI) was performed, which showed venous thrombosis in the right transverse sinus, bilateral sigmoid sinuses, and bilateral internal jugular veins. These findings, combined with the patient’s binocular diplopia, suggest an increased intracranial pressure secondary to CVST.

**Diagnoses:**

The patient was diagnosed with thrombocytopenia secondary to APS complicated by extensive CVST.

**Interventions:**

The patient received intravenous immunoglobulin, methylprednisolone pulse therapy, and rituximab to treat immune thrombocytopenia. She accepted retrograde balloon-assisted venous thrombectomy for CVST.

**Outcomes:**

During the 1-year follow-up, the patient did not experience a decrease in platelet count or recurrence of thrombosis while being maintained on anticoagulants and immunosuppressants.

**Lessons:**

This case highlights that endovascular thrombectomy (EVT) may be a rescue option for CVST in patients with triple-positive antiphospholipid syndrome and severe thrombocytopenia, once platelet counts are stabilized with immunosuppressive therapy. Prompt correction of thrombocytopenia can create a safe window for intervention, potentially avoiding neurological deterioration when anticoagulation alone is insufficient or is contraindicated.

## 1. Introduction

Cerebral venous sinus thrombosis (CVST) is a rare cerebrovascular disorder that accounts for 0.5% to 3% of all stroke cases.^[1]^ The most common occlusion sites are the transverse and superior sagittal sinuses, and occlusion results in impaired venous return, cerebral edema, hemorrhagic infarction, or elevated intracranial pressure.^[1,2]^ Clinically, patients with CVST present with headaches (88.8%), seizures (39.3%), paresis (37.2%), papilledema (28.3%), and altered mental status (22%).^[2,3]^ The acute-phase in-hospital mortality rate ranges from 1% to 4%.^[4]^ Most patients achieve functional independence after treatment; however, residual symptoms (e.g., cognitive impairment, mood disturbances, fatigue, headaches, and seizures) may persist, particularly in patients with cerebellar hemorrhagic lesions.^[1]^ CVST predominantly affects young women, with nearly half of the cases being linked to oral estrogen use or pregnancy.^[5]^ Other risk factors include antiphospholipid antibody syndrome (APS), *JAK2* mutations, malignancy, and systemic lupus erythematosus (SLE).^[1]^ Among the different thrombophilia conditions that warrant special attention, patients with APS and SLE have a high risk of thrombosis and phlebitis, which may be attributed to the immune complex deposition, endothelial dysfunction, and coagulopathy due to APS or a concomitant nephrotic syndrome.^[5–7]^

Standard treatment approaches include oral anticoagulation for 3 to 6 months. Endovascular therapy (EVT) serves as rescue therapy for patients with thrombus progression under anticoagulants and those with contraindications to anticoagulation.^[1,8,9]^ Despite the growing use of EVT in selected CVST cases, evidence of its safety and efficacy in patients with both severe thrombocytopenia and autoimmune disorders, such as APS and SLE, remains limited. In this context, we present a case of CVST secondary to APS and SLE complicated by severe thrombocytopenia that was successfully treated with balloon-assisted thrombosuction after therapy with high-dose glucocorticoids, immunosuppressants, intravenous immunoglobulin (IVIG), and rituximab for thrombocytopenia.

## 2. Case presentation

A 33-year-old Taiwanese woman presented to the emergency room (ER) with a 2-day history of fever, headache, and dizziness. The headache improved partially after acetaminophen, but intermittent fever persisted. The patient was then transferred to the ward on the same day. Three days before the ER visit, she was discharged from the chest ward with a newly diagnosed SLE based on hemolytic anemia, proteinuria (urine protein–creatinine ratio: 9816 mg/g), pleural effusion, antinuclear antibody positivity, and hypocomplementemia. She was an office worker and there were no other relevant comorbidities or family history. She denied other associated symptoms, including shortness of breath, chest tightness, muscle weakness, numbness, tinnitus, melena, or changes in bladder or bowel habits. Physical examination revealed a body height of 155 cm, body weight of 40 kg, temperature of 36.8 °C, pulse of 68 beats/min, blood pressure of 103/78 mm Hg, and respiratory rate of 16 breaths/min. Despite low body mass index and ill-looking, she had adequate nutrition intake and good spirit. Complete blood counts in the ER revealed a white blood cell count of 7410/µL, hemoglobin level of 10.1 g/dL, and platelet count of 17,000/μL Her serum creatinine level was 1.22 mg/dL with positive ANA (1:80, AC-2 pattern) and hypocomplementemia (C3, 82 mg/dL; C4, 11 mg/d). However, anti-dsDNA, anti-Sm, anti-RNP, anti-Ro/La were all negative. Chest radiography revealed consolidation of the left lung.

**Figure 1.**
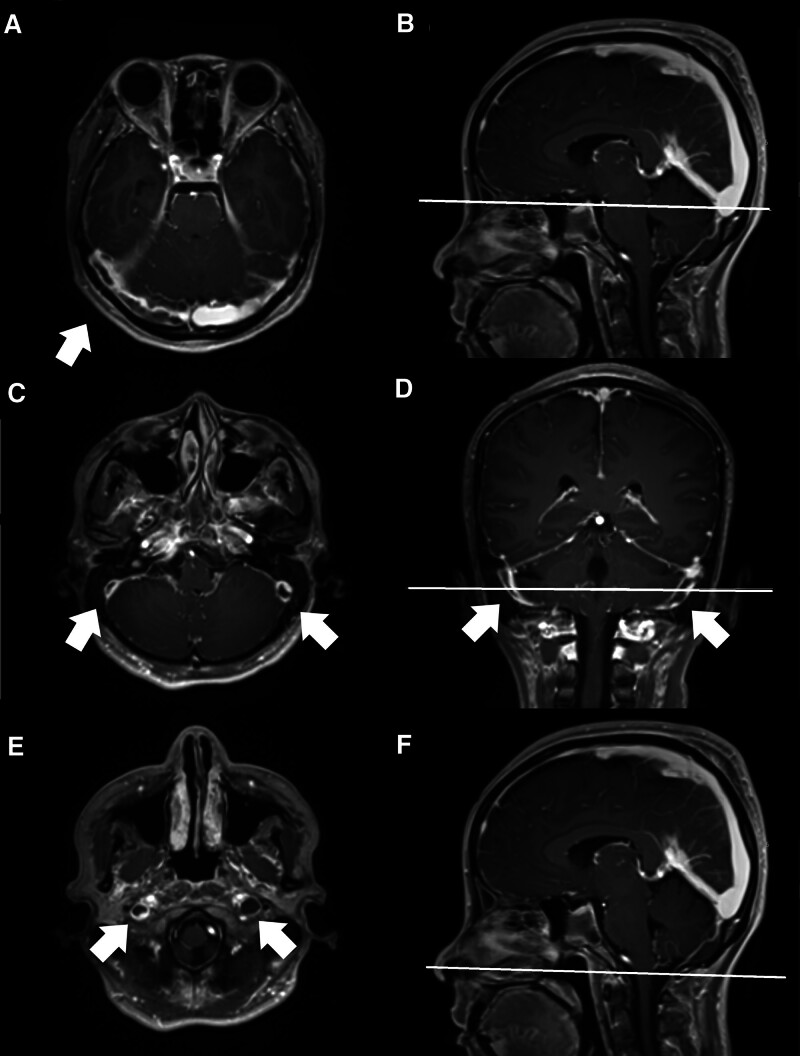
MRI scan of brain T1 image with contrast medium of a 33-yr-old woman reveals cerebral venous sinus thrombosis in the right transverse sinus, bilateral sigmoid sinuses, and bilateral internal jugular veins. (A) A transverse view of the brain MRI demonstrates a thrombus in the right transverse sinus. (B) A sagittal view of the brain MRI shows a hyperintense signal in the superior sagittal and straight sinuses. The white line indicates the image level of (A). (C) A transverse view of the brain MRI demonstrates the absence of flow in the bilateral sigmoid sinuses. (D) A coronal view of the brain MRI shows a thrombus in the bilateral sigmoid sinuses. The white line indicates the image level of (C). (E) A transverse view of the brain MRI demonstrates a thrombus in the bilateral internal jugular vein. (F) The transverse line in the sagittal view of the brain MRI scan indicates the image level of (E). MRI = magnetic resonance image.

Following admission, empirical ceftriaxone was initiated under the suspicion of pneumonia. The patient was diagnosed with immune thrombocytopenic purpura after ruling out thrombotic thrombocytopenic purpura, given the normal level of a disintegrin and metalloproteinase with thrombospondin type 1 motif member 13 (79%). The autoimmune profile was positive for lupus anticoagulant (2.01, >2.0 strongly present) and showed high titers of anticardiolipin IgG (78 U/mL) and anti-β2-glycoprotein IgG (66 EU/mL), indicating a triple-positive APS profile. Considering the low body weight of this patient, methylprednisolone pulse therapy (500 mg) was administered from day five to seven and followed by intravenous methylprednisolone 20 mg every 12 hours under the impression of immune thrombocytopenia and acute kidney injury due to suspected lupus nephritis. No angiotensin-converting enzyme inhibitors or angiotensin II receptor blocker (ARB) was given due to elevated creatinine and low systolic blood pressure. She was administered oral hydroxychloroquine (200 mg) daily and B-cell depletion therapy with 500 mg rituximab on day five. Right periorbital ecchymosis was also noted on hospital day five, and the platelet count was 14,000/μL on the following day. On day nine of hospitalization, the patient developed a new-onset diplopia. Her consciousness was clear with a Glasgow Coma Scale score of E4V5M6. Physical examination revealed an impaired right lateral gaze with an inability to fully abduct the right eye, consistent with right abducens nerve palsy. Fundoscopic examination of the right eye revealed mild blurring of the nasal disc margins, suggesting increased intracranial pressure. Brain magnetic resonance imaging (MRI) performed on the same day revealed venous thrombosis in the right transverse (Fig. 1A and B) and bilateral sigmoid sinuses (Fig. 1C and D), and bilateral internal jugular veins (Fig. 1E and F). These findings, combined with the patient’s binocular diplopia, suggested increased intracranial pressure secondary to cerebral venous thrombosis. Concomitant APS and SLE were also confirmed.

**Figure 2.**
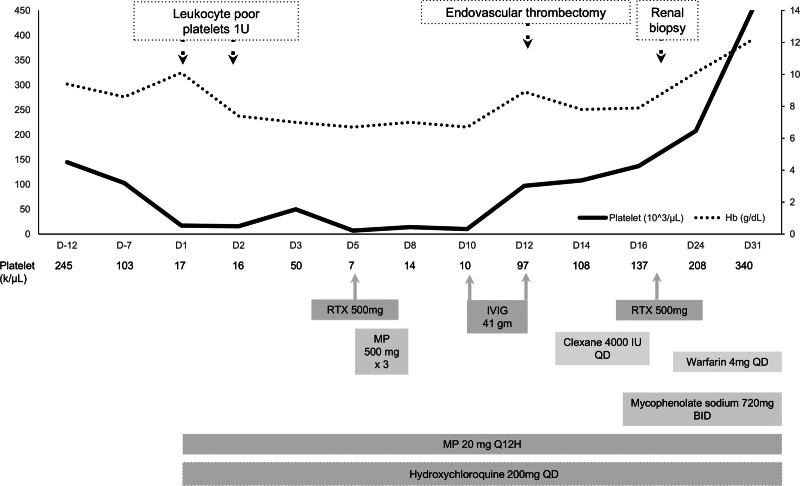
Timeline of the therapy for thrombocytopenia and the invasive intervention, including endovascular sinus thrombectomy and renal biopsy after thrombocytopenia improvement. Exact platelet counts (k/μL) are labeled below horizontal axis at each displayed time-point. Open boxes and dashed arrows indicate platelet transfusion, endovascular intervention, and renal biopsy. Grey boxes and arrows indicate the timing of medical treatment. QD = daily, BID = twice daily, IVIG = intravenous immunoglobulin, MP = methylprednisolone, RTX = rituximab.

**Figure 3.**
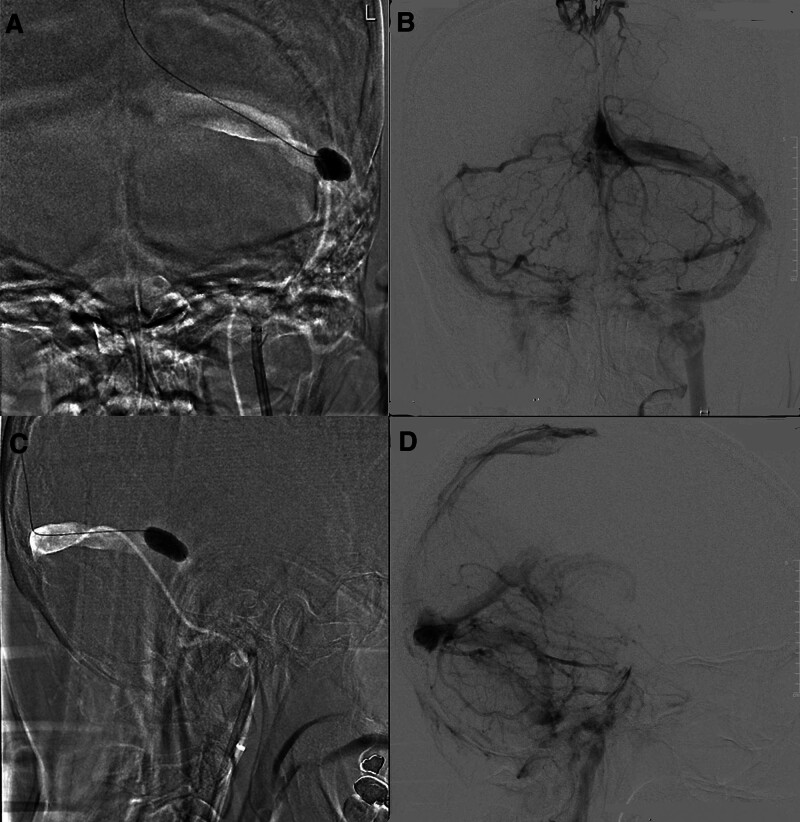
Retrograde venous thrombectomy and post-thrombectomy venography. (A) Coronal view of venous thrombectomy with balloon-assisted thrombosuction. (B) Coronary angiography after thrombectomy shows passage of contrast into the bilateral transverse sinus. (C) Sagittal view of venous thrombectomy with balloon-assisted thrombosuction. (D) Sagittal angiography after thrombectomy showing recanalization of the left sigmoid sinus and internal jugular vein, without venous retention of the left transverse sinus.

On hospital day 10, the patient’s platelet count was 10,000/μL despite receiving glucocorticoid pulse therapy and rituximab. Given the refractory immune thrombocytopenia and the high risk of life-threatening intracranial hemorrhage, total IVIG 1 g/kg was administrated in divided dose on days 10 and 11 (Fig. 2). After IVIG treatment, the platelet count improved from 10,000/μL to 97,000/μL on day 12. On the same day, percutaneous transluminal angiography of the brain via the left femoral artery revealed thrombosis in the right transverse sinus, bilateral sigmoid sinuses, and bilateral internal jugular veins. Retrograde balloon-assisted venous thrombectomy was performed via the right femoral vein using a NeuronMAX 088 guiding sheath, 4-Fr. JB2 diagnostic catheter, Contata 028 microcatheter system, and HyperForm 7 × 15-mm balloon catheter. Postprocedural angiography revealed improved recanalization of the sigmoid sinus and internal jugular vein, without significant venous stagnation (Fig. 3). Her blood pressure was 122/82 mm Hg before and 113/85 mm Hg after the procedure. Thrombus pathology revealed no abnormal findings.

The patient’s binocular diplopia improved immediately after the intervention, and anticoagulation with enoxaparin (4000 IU) was administered the following day (day 13). Enoxaparin was discontinued on day 17, and renal biopsy was performed the next day. A second dose of rituximab (500 mg) was administered on day 18. Renal biopsy confirmed lupus nephritis, class IV-S (C). Oral warfarin (4 mg/day) was administered after the renal biopsy. Panendoscopy performed to determine the cause of anemia revealed hemorrhagic gastropathy and reflux esophagitis; therefore, esomeprazole was prescribed. The patient was discharged on day 19 in stable condition with prednisolone 10 mg twice daily. Eight-month follow-up brain MRI showed complete venous recanalization. A summary of the treatment course is presented in Figure 2. During the 1-year follow-up, the patient did not experience a decrease in platelet count or recurrence of thrombosis while being maintained on anticoagulants and immunosuppressants. One year after this admission, proteinuria decreased to 792 mg/g and maintenance prednisolone dose was 10 mg daily. She felt satisfied with the clinical outcomes of neurological, hematological, and nephrological manifestations (Table 1).

**Table 1:** Laboratory results of this admission and follow-ups in this patient.

	Admission	1 mo later	8 mo later	1 yr later	Reference range
Haemoglobin (g/dL)	10.1	12.2	13.2	13.7	12.0–16.0
White blood cell count (/μL)	7410	9850	6600	9090	3800–10,400
Platelets (k/μL)	17	340	315	297	140–400
INR	1.13	2.24	1.64	1.5	0.8–1.2
BUN (mg/dL)	24.0	32.0	10.0	NA	7.0–25.0
Creatinine (mg/dL)	1.22	0.82	0.63	0.77	0.4–1.5
UPCR (mg/g)	9816	2835	1400	792	<200
Albumin (g/dL)	2.1	3.0	3.9	3.7	3.5–5.7
C3 (mg/dL)	82.6	135.9	103.1	107.6	87.0–200.0
C4 (mg/dL)	11.0	23.7	21.7	21.1	19.0–52.0
Anti-dsDNA (IU/ml)	5.50	1.70	5.30	1.4	<10
ESR (mm/hr)	106	50	6	3	0–20
CRP (mg/dL)	5.153	0.148	NA	NA	0–1.0
Anti-Cardiolipin IgG (GPL-U/mL)	78.0	NA	6.9	3.9	<10.0
Anti-B2-GP1 IgG (EU/mL)	66.0	23.0	3.8	5.3	0.0–7.0
Lupus anti-coagulant (dRVVT)	2.09	NA	NA	NA	<1.20 factor deficiency 1.20–1.50 weakly present 1.51–2.00 moderately present >2.00 strongly present

Anti-dsDNA = anti-double-stranded deoxyribonucleic acid, BUN = Blood urea nitrogen, C3 = complement component 3, C4 = complement component 4, CRP = C-reactive protein, dRVVT = diluted Russell’s viper venom time, NA = not applicable, UPCR = urine protein and creatinine ratio.

## 3. Discussion

This case report demonstrates the successful use of balloon-assisted thrombosuction for CVST in a patient recovering from severe thrombocytopenia after treatment with corticosteroid pulse therapy, anti-CD20 monoclonal antibody (rituximab), and IVIG. We chose two doses of rituximab 500 mg for thrombocytopenia due to the patient’s low body weight and financial status. IVIG was given for its faster onset in thrombocytopenia, although the benefit of IVIG should be balanced by the risk of increased viscosity and potential thrombosis risk. Nephrotic syndrome is a risk factor of thrombosis, therefore renal biopsy for a definite diagnosis and treatment is essential. The best practice for nephrotic syndrome is to initiate angiotensin-converting enzyme inhibitors or ARB. However, due to acute renal function deterioration and brain sinus thrombosis with impaired brain perfusion, these treatments were not applied in this patient.

Considering the patient’s risk of bleeding from immune thrombocytopenia, low molecular weight heparin and anticoagulants were cautiously prescribed for brain sinus thrombosis. Traditional thrombolytic therapy is unlikely to resolve multiple sinus thromboses in such cases. Therefore, endovascular intervention was performed, as it is the recommended second-line therapy for patients with CVST presenting with clinical deterioration despite anticoagulation therapy, those with progression of intracerebral hemorrhage and venous infarction, or those with contraindications to anticoagulation (e.g., thrombocytopenia < 100,000/μL or recent gastrointestinal bleeding).^[10,11]^ Considering the triple-positive antiphospholipid syndrome status, we prescribed a vitamin K antagonist as a long-term anticoagulant treatment for APS secondary thromboprophylaxis, with a target of INR 2 to 3.

Previous studies have shown that anticardiolipin antibodies are associated with CVST, particularly when combined with triggering factors such as oral contraceptive use, pregnancy, and puerperium.^[12,13]^ A case series with review of the literature analyzed 113 APS cases with CVST and found that only 5% of the patients presented with triple-positive APS profile. The most frequently detected laboratory abnormality was anticardiolipin (66%), followed by lupus anticoagulant (56%) and anti-β_2-_glycoprotein I antibody (11%).^[14]^ Five patients received additional thrombolysis therapy, while four patients with complicated disease courses underwent surgical interventions, including decompressive craniectomy, ventriculoperitoneal shunt placement, and fistula embolization. No EVT intervention was documented in this study. Sakamoto et al reported a patient with APS who presented with cerebral sinus thrombosis and deep vein thrombosis, which exacerbated despite adequate anticoagulant therapy. Catheter-directed thrombolysis also failed to improve his neurological manifestations. Finally, optic canal decompression and cistern-peritoneal shunt operation were performed, indicating the complexity of the treatment for CVST.^[15]^ Briefly, CVST may occur in patients with APS-associated thrombocytopenia; however, the use of EVT has rarely been reported.

Therefore, we performed a PubMed search using the terms “(cerebral venous sinus thrombosis) AND (antiphospholipid) AND/OR (endovascular intervention)” to identify articles on CVST associated with APS. In a retrospective study by Shen et al., 4 of 21 patients received endovascular mechanical thrombectomy therapy; of these, three achieved a good prognosis and one died.^[16]^ However, platelet counts at the time of the intervention were not available in this study. EVT is not considered a first-line treatment for CVST because routine EVT has not shown clinical benefits compared to standard anticoagulation therapy;^[1]^ however, EVT may play a role as a rescue therapy in complicated cases. Zhang et al demonstrated the efficacy of endovascular therapy. They reported complete recanalization in six of ten patients who underwent sliding balloon-assisted thrombectomy combined with intrasinus thrombolysis, with no procedure-related complications at the 6-month follow-up.^[8]^ Prospective studies of patients with CVST undergoing EVT have identified favorable prognostic factors, including younger age, absence of hemorrhagic venous infarction, noninvolvement of the frontoparietal lobes and superior sagittal sinus, and short thrombus length.^[17,18]^ In sum, only a few cases of APS-associated CVST treated with endovascular therapy have been reported.^[16]^ These studies suggest that EVT remains a technically demanding procedure but may serve as a rescue therapy.

Thrombocytopenia is a critical concern in CVST management. However, clinical studies of this topic are lacking compared to arterial intervention. Thrombocytopenia is associated with increased mortality after mechanical thrombectomy in patients with ischemic stroke due to large vessel occlusion, defined as proximal vessel obstruction, including the internal carotid artery, proximal anterior, middle, and posterior cerebral arteries, intracranial vertebral artery, and basilar artery.^[19]^ The RESCUE-Japan Registry 2 study in patients with large vessel occlusion requiring EVT reported thrombocytopenia (platelet count < 150,000/µL) in 18.4% of cases and examined the association between platelet and clinical outcome.^[20]^ The odds ratios (ORs) of mortality were 2.76 and 3.26 in the mild thrombocytopenic (platelet 100,000–150,000/µL) and moderate/severe groups (platelet < 100,000/µL), respectively, compared with the control group. Symptomatic intracranial hemorrhage increased with the severity of thrombocytopenia, and adjusted odds ratios were 4.43 (1.16–17.0) in moderate/severe and 1.85 (0.71–4.86) in mild thrombocytopenia. Therefore, consider the lower risk of bleeding in venous than arterial intervention, platelet count of 100,000/ul may be a reasonable target for safety requirement. Further studies are required to confirm these findings. Thrombocytopenia occurs in 20%–50% of patients with APS.^[21,22]^ Moreover, for severe thrombocytopenia (platelet count < 50,000/µL) requiring anticoagulation, full-dose anticoagulant therapy is recommended once the platelet count is > 50,000/µL after systemic steroid and IVIG treatment.^[23]^ IVIG is typically administered in cases of refractory thrombocytopenia, and its therapeutic mechanisms include inhibition of Fc-receptor-mediated platelet phagocytosis, suppression of antiplatelet antibody production, and anti-idiotypic inhibition of antiplatelet antibodies.^[24]^ Although the optimal platelet count for venous endovascular intervention remains unclear, our case demonstrates that timely treatment of thrombocytopenia to a level of 100,000/ul before elective endovascular intervention is a practical goal for reducing bleeding complications. However, this single-center case lacks a control group and cannot establish universal guidance on the optimal timing or safe platelet threshold for EVT in APS-related CVST with severe thrombocytopenia. Larger, multicenter studies are warranted.

## 4. Conclusion

Despite the uncertainty regarding safe platelet counts before starting the intervention, EVT immediately after thrombocytopenia correction may prevent thrombosis progression and bleeding owing to venous congestion in patients with CVST and thrombocytopenia. Compared to concurrent immunosuppressants for thrombocytopenia and anticoagulants alone for thrombosis, our approach, which incorporates additional EVT, may reduce uncertainty in treatment and shorten the duration of hospitalization. Although this report focused on a single patient, limiting the generalizability of the findings, it highlights the need for further research to determine the standardized criteria for the timing of EVT and the minimum safe platelet count threshold to guide clinical decision-making in such scenarios.

## Acknowledgments

We extend our gratitude to Far Eastern Medical Hospital for their generous assistance.

## Author contributions

**Conceptualization:** Yu Chen, Chien-Sheng Wu.

**Data curation:** Yen-Jun Lai, Ting-Hui Chang.

**Formal analysis:** Yu Chen, Yen-Jun Lai, Ting-Hui Chang, Chien-Sheng Wu.

**Methodology:** Yen-Jun Lai.

**Supervision:** Chien-Sheng Wu.

**Validation:** Ting-Hui Chang, Chien-Sheng Wu.

**Writing – original draft:** Yu Chen.

**Writing – review & editing:** Yu Chen, Chien-Sheng Wu.

## References

[1] Saposnik G Bushnell C Coutinho JM; American Heart Association Stroke Council; Council on Cardiopulmonary, Critical Care, Perioperative and Resuscitation; Council on Cardiovascular and Stroke Nursing; and Council on Hypertension. Diagnosis and management of cerebral venous thrombosis: a scientific statement from the American Heart Association. Stroke. 2024;55:e77–90. 38284265 10.1161/STR.0000000000000456

[2] Ferro JM Canhao P Stam J Bousser M-G Barinagarrementeria F; ISCVT Investigators. Prognosis of cerebral vein and dural sinus thrombosis: results of the International Study on Cerebral Vein and Dural Sinus Thrombosis (ISCVT). Stroke. 2004;35:664–70. 14976332 10.1161/01.STR.0000117571.76197.26

[3] Capecchi M Abbattista M Martinelli I. Cerebral venous sinus thrombosis. J Thromb Haemost. 2018;16:1918–31. 29923367 10.1111/jth.14210

[4] Rezoagli E Bonaventura A Coutinho JM. Incidence rates and case-fatality rates of cerebral vein thrombosis: a population-based study. Stroke. 2021;52:3578–85. 34372672 10.1161/STROKEAHA.121.034202 PMC10082066

[5] Leal Rato M Bandeira M Romao VC Aguiar de Sousa D. Neurologic manifestations of the antiphospholipid syndrome – an update. Curr Neurol Neurosci Rep. 2021;21:41. 34125304 10.1007/s11910-021-01124-z PMC8200381

[6] Zhang B Lang Y Zhang W Cui Li Deng F. Characteristics and management of autoimmune disease-associated cerebral venous sinus thrombosis. Front Immunol. 2021;12:671101. 34367137 10.3389/fimmu.2021.671101 PMC8339549

[7] Sayar Z Moll R Isenberg D Cohen H. Thrombotic antiphospholipid syndrome: a practical guide to diagnosis and management. Thromb Res. 2021;198:213–21. 33485122 10.1016/j.thromres.2020.10.010 PMC7560059

[8] Zhang M Jiang F Wen Q. Sliding balloon-assisted thrombectomy combined with aspiration and intrasinus urokinase thrombolysis for the treatment of hemorrhagic cerebral venous sinus thrombosis: experience of 10 patients. Front Neurol. 2025;16:1519308. 40125396 10.3389/fneur.2025.1519308 PMC11925791

[9] Burns JD Orru E. Endovascular therapy for severe cerebral venous sinus thrombosis: time is vein? Neurocrit Care. 2024;41:728–9. 39042278 10.1007/s12028-024-02045-8

[10] Lee SK Mokin M Hetts SW Fifi JT Bousser M-G Fraser JF; Society of NeuroInterventional Surgery. Current endovascular strategies for cerebral venous thrombosis: report of the SNIS Standards and Guidelines Committee. J Neurointerv Surg. 2018;10:803–10. 29871990 10.1136/neurintsurg-2018-013973

[11] Siddiqui FM Weber MW Dandapat S. Endovascular thrombolysis or thrombectomy for cerebral venous thrombosis: study of nationwide inpatient sample 2004-2014. J Stroke Cerebrovasc Dis. 2019;28:1440–7. 30952531 10.1016/j.jstrokecerebrovasdis.2019.03.025

[12] Christopher R Nagaraja D Dixit NS Narayanan CP. Anticardiolipin antibodies: a study in cerebral venous thrombosis. Acta Neurol Scand. 1999;99:121–4. 10071172 10.1111/j.1600-0404.1999.tb00669.x

[13] Carhuapoma JR Mitsias P Levine SR. Cerebral venous thrombosis and anticardiolipin antibodies. Stroke. 1997;28:2363–9. 9412615 10.1161/01.str.28.12.2363

[14] Jerez-Lienas A Mathian A Aboab J. Cerebral vein thrombosis in the antiphospholipid syndrome: analysis of a series of 27 patients and review of the literature. Brain Sci. 2021;11:1641. 34942943 10.3390/brainsci11121641 PMC8699363

[15] Sakamoto S Akutsu K Kawase K. Simultaneous presentations of deep vein thrombosis and cerebral sinus thrombosis in a case of primary antiphospholipid syndrome. Angiology. 2008;59:765–8. 18388066 10.1177/0003319707309310

[16] Shen H Huang X Fan C. Clinical characteristics and management of cerebral venous sinus thrombosis in patients with antiphospholipid syndrome: a single-center retrospective study. Clin Appl Thromb Hemost. 2021;27:1076029621999104. 33872100 10.1177/1076029621999104 PMC8058809

[17] Alwan A Miraclin AT Bal D. Management of severe cerebral venous sinus thrombosis using mechanical balloon assisted thrombectomy. Stroke: Vasc Intervent Neurol. 2023;3:e000574. 10.1161/SVIN.122.000574 PMC12778598 41585770

[18] Liao CH Liao NC Chen WH. Endovascular mechanical thrombectomy and on-site chemical thrombolysis for severe cerebral venous sinus thrombosis. Sci Rep. 2020;10:4937. 32188921 10.1038/s41598-020-61884-5 PMC7080812

[19] Domingo RA Tripathi S Perez-Vega C. Influence of platelet count on procedure-related outcomes after mechanical thrombectomy for large vessel occlusion: a systematic review and meta-analysis. World Neurosurg. 2022;157:187–92.e1. 34653708 10.1016/j.wneu.2021.10.080

[20] Fujiwara S Sakai N Imamura H; RESCUE-Japan Registry 2 Investigators. Impact of thrombocytopenia on hemorrhagic complications after endovascular therapy for acute large vessel occlusion: sub-analysis of RESCUE-Japan registry 2. J Neurol Sci. 2023;449:120659. 37079972 10.1016/j.jns.2023.120659

[21] Cervera R Serrano R Pons-Estel GJ; Euro-Phospholipid Project Group (European Forum on Antiphospholipid Antibodies). Morbidity and mortality in the antiphospholipid syndrome during a 10-year period: a multicentre prospective study of 1000 patients. Ann Rheum Dis. 2015;74:1011–8. 24464962 10.1136/annrheumdis-2013-204838

[22] Tomasello R Giordano G Romano F. Immune thrombocytopenia in antiphospholipid syndrome: is it primary or secondary? Biomedicines. 2021;9:1170. 34572358 10.3390/biomedicines9091170 PMC8472578

[23] Matzdorff A Beer JH. Immune thrombocytopenia patients requiring anticoagulation--maneuvering between Scylla and Charybdis. Semin Hematol. 2013;50(Suppl 1):S83–8. 23664524 10.1053/j.seminhematol.2013.03.020

[24] Hansen RJ Balthasar JP. Mechanisms of IVIG action in immune thrombocytopenic purpura. Clin Lab. 2004;50:133–40. 15074465

